# Euglycemic Ketoacidosis Following the Use of Counterfeit Semaglutide for Weight Loss

**DOI:** 10.7759/cureus.102627

**Published:** 2026-01-30

**Authors:** Mira Sterckx, Ludo De Keyser

**Affiliations:** 1 Internal Medicine, University of Antwerp, Antwerpen, BEL; 2 Endocrinology and Diabetes, ZAS (Ziekenhuis aan de Stroom) Cadix, Antwerp, BEL

**Keywords:** euglycemic diabetic ketoacidosis, glp-1 agonist, off-label drug use, semaglutide, treating obesity

## Abstract

Euglycemic ketoacidosis is a rare but increasingly recognized adverse effect related to the use of glucagon-like peptide-1 (GLP-1) receptor agonists. Reduced oral intake in combination with gastrointestinal side effects can trigger a starvation state and ketone body formation. The growing off-label use of glucagon-like peptide-1 (GLP-1) agents for weight loss, particularly without medical supervision, may further increase the risk of this serious complication. An 18-year-old woman with no medical history presented with persistent nausea, intractable vomiting, and reduced oral intake for three days. Ten days earlier, she had initiated semaglutide purchased online and self-administered escalating doses without supervision. Laboratory evaluation revealed high-anion gap metabolic acidosis (pH 7.24, bicarbonate 14 mmol/L, anion gap 24 mEq/L), markedly elevated β-hydroxybutyrate (5.9 mmol/L), and normoglycemia (60 mg/dL), consistent with euglycemic ketoacidosis. She improved rapidly with intravenous fluids, dextrose infusion, and supportive therapy and was discharged in stable condition after 36 hours. This case highlights an uncommon but serious adverse condition observed in association with semaglutide use in a non-diabetic patient. Contributing factors include possible inappropriate high-dose initiation and uncertainties in dosing and purity related to unregulated online products. Clinicians should be aware of euglycemic ketoacidosis as a potential complication of GLP-1 receptor agonists, particularly when obtained through unsafe sources and used without proper medical monitoring.

## Introduction

With the increasing prevalence of obesity, glucagon-like peptide-1 (GLP-1) medications have emerged as highly effective agents for weight reduction. While these medications are generally considered safe, their expanding use has been accompanied by growing concern regarding both established and emerging adverse effects. This concern is further heightened by the increasing use of semaglutide obtained from unregulated online pharmacies, where counterfeit or non-quality-controlled products may increase the risk of unintended and potentially serious adverse effects.

A clinical review assessing tolerability, side effects, and risks of liraglutide, semaglutide, and tirzepatide showed that the most side effects were gastrointestinal related (nausea, diarrhea, and constipation), whereby the majority were considered mild-moderate and transient. More serious, but less common, side effects reported include pancreatitis, bowel obstruction, and gastroparesis [1]. In contrast, ketoacidosis represents a rare and emerging potential complication that is not yet well characterized within the overall safety profile of GLP-1 receptor agonists, particularly in patients without diabetes.

Ketoacidosis is a potentially life-threatening metabolic state caused by the accumulation of ketones. Diabetic ketoacidosis is the most common and widely recognized form; however, ketoacidosis can also occur in the absence of diabetes. Euglycemic ketoacidosis is defined as a glycemia <200 mg/dL, high anion gap metabolic acidosis, and ketosis [2]. The absence of hyperglycemia can delay proper diagnosis and treatment. Reported etiologies include starvation ketoacidosis, alcoholic ketoacidosis, and sodium glucose-linked transporter type 2 inhibitor (SGLT2i) ketoacidosis. Physiological states such as pregnancy or acute illness precipitate starvation ketoacidosis due to increased insulin resistance combined with reduced caloric intake from nausea and vomiting, resulting in a more rapid progression of metabolic disturbance [2,3].

Regarding the use of GLP-1 receptor agonists for off-label obesity management, ketoacidosis is an uncommon but previously associated complication. A recent brief review in March 2025 by Sood et al. summarized the three published case reports on the topic to date [4].

In this case, we discuss a patient without diabetes taking semaglutide for weight loss purposes, presenting with euglycemic ketoacidosis. Notably, she was taking it without medical supervision after acquiring the medication from an online pharmacy. This raises further concern for counterfeit or possibly improperly dosed semaglutide, which could also contribute to adverse metabolic effects. In this present case, starvation is proposed as the most likely mechanism underlying the development of euglycemic ketoacidosis.

## Case presentation

An 18-year-old woman with no significant past medical history presented to the emergency department with a three-day history of persistent nausea and intractable vomiting. Ten days prior to presentation, she had initiated subcutaneous semaglutide, which she had obtained online without medical supervision. Relying on non-verified information found on the Internet, she self-administered 30 “clicks” of a 1-mg labelled semaglutide pen for her first dose and increased this to 36 clicks for a second dose administered three days prior to admission. However, estimating the administered dose based on the number of “clicks” is inherently unreliable, particularly given the uncertain authenticity and calibration of the product. Dose-estimation assumes standard device calibration, suggesting that 30 clicks would correspond to 0.5 mg, yet the actual delivered dose remains unknown and cannot be reliably determined.

The patient reported mild nausea beginning shortly after the initial injection, which progressed to continuous vomiting following the second injection. Over the next three days, she was unable to tolerate any solid food and consumed only minimal fluids. She also noted a marked reduction in urine output, describing only a few drops over the previous 48 hours, and reported no bowel movements during this period. She denied any fever, abdominal pain, or diarrhea. She was not taking any other medications. She denied alcohol use. Clinical examination revealed stable vital signs, including a blood pressure of 126/92 mmHg, heart rate of 87 beats per minute, oxygen saturation of 97% on room air, and a temperature of 36.3°C. Physical assessment noted signs of dehydration, including reduced skin turgor and dry oral mucosa.

At baseline, the patient weighed 65 kg with a BMI of 26.4 kg/m² (height 157 cm). On presentation, her weight had decreased to 60 kg, corresponding to a BMI of 24.3 kg/m² and representing a weight loss of 5 kg over 10 days.

Diagnostic evaluation

Initial laboratory studies demonstrated a high-anion gap metabolic acidosis with a venous pH of 7.24, serum bicarbonate of 14 mmol/L, and an anion gap of 24 mEq/L. Capillary β-hydroxybutyrate was markedly elevated at 5.9 mmol/L (reference <0.5 mmol/L), consistent with significant ketosis. The patient's serum glucose was 60 mg/dL, representing a normal glucose level. In non-diabetic patients, in the absence of symptoms, hypoglycemia is only considered when serum glucose is <55 mg/dL [5]. Hemoglobin was elevated at 15.4 g/dL, suggestive of hemoconcentration due to dehydration. Serum creatinine remained within normal limits at 0.62 mg/dL (baseline 0.54 mg/dL), indicating preserved renal function despite markedly reduced oral intake. C-reactive protein (CRP) was low at 2.6 mg/L. According to these laboratory results, there was no indication of an underlying acute condition. A negative ethanolemia with no history of alcohol use made alcohol related ketoacidosis unlikely. A negative human chorionic gonadotropin (hCG) ruled out pregnancy. Laboratory test results are given in Table 1. In the presence of high-anion gap metabolic acidosis, marked ketonemia, normal serum glucose, and a history of prolonged nausea and vomiting leading to reduced oral intake, the findings were consistent with euglycemic starvation ketoacidosis.

**Table 1:** Laboratory results Bolded labs indicate abnormal results. Blank results were not measured at that time. ALT: alanine aminotransferase; CRP: C-reactive protein; hCG: human chorionic gonadotropin

Test	Patient Value (0 hours)	Patient Value (+2 hours)	Patient Value (+20 hours)	Reference Range
Venous pH	7.24	7.35	7.39	7.32–7.43
Anion gap	24	21	9	10-18 mEq/L
Lactic acid	1.7	1.3	0.9	0.7–2.5 mmol/L
Glucose	60	93	103	74-106 mg/dL
Sodium	137	136	140	136-145 mmol/L
Potassium	4.0	3.4	3.1	3.5-5.0 mmol/L
Bicarbonate	14	17	26	22-29 mmol/L
Capillary β-hydroxybutyrate	5.9	-	1.1	<0.5 mmol/L
Hemoglobin	15.4	12.9	12.4	11.2-15.1 g/dL
Creatinine	0.62	-	0.48	0.51 – 0.95 mg/dL
Bilirubin	0.6	-	0.4	<1.2 mg/dL
ALT	10	-	10	<35 U/L
Lipase	26	-	-	13-60 U/L
CRP	2.6	-	2	<5 mg/L
hCG	<5	-	-	<5IU/L
Ethanolemia	<0.1	-	-	<0,1g/L

Treatment

Treatment was initiated between presentation (0 hours) and +2 hours in the emergency department. The patient was treated with 1,000 mL of PlasmaLyte (isotonic crystalloid; Baxter International Inc., Deerfield, Illinois, United States), 100 mL of 8.4% sodium bicarbonate solution (equivalent to 100 mEq), and 50 mg of alizapride for antiemetic therapy. Although bicarbonate therapy is generally reserved for severe metabolic acidosis (pH < 7.1), its administration in this case was a clinically directed decision made in the emergency department. Following initial resuscitation, a continuous infusion was initiated consisting of PlasmaLyte at 42 mL/hour and 5% dextrose at 84 mL/hour. She subsequently tolerated small amounts of oral intake. After 24 hours, her nausea and vomiting had resolved, and she was able to resume a regular diet, allowing discontinuation of intravenous fluids. Rapid metabolic improvement with normalisation of acidosis seen at +2 hours after receiving dextrose-containing fluids further supports the diagnosis of starvation keto-acidosis. Furthermore, laboratory evaluation at +2 hours demonstrated a mild hypokalemia of 3.4 mmol/L, which progressed to 3.1 mmol/L after +20 hours. The absence of significant hypokalemia at admission was likely due to extracellular potassium shift in the context of metabolic acidosis. With correction of the acidosis, potassium shifted intracellularly, unmasking an underlying total body potassium deficit. Given the patient’s clinical improvement, adequate oral intake, and resolution of vomiting, spontaneous normalization of serum potassium was anticipated after discharge; therefore, potassium supplementation was not deemed necessary. The patient was discharged in stable condition after a total hospital stay of 36 hours.

Outcome and follow-up

The patient was advised to discontinue semaglutide. The dangers of unauthorized medication without follow-up were discussed with her. She received a consultation with a dietician during her stay. A psychological consult was scheduled as an outpatient appointment.

## Discussion

GLP-1 agonists such as liraglutide, semaglutide, or tirzepatide were initially developed for the treatment of type 2 diabetes. Since then, studies have proved success for its off-label use in obesity [6,7]. Side effects are usually mild-moderate, whereby tolerability increases over time [1]. The occurrence of euglycemic keto-acidosis in patients without diabetes using GLP-1 agonists has previously been described in three case reports [8-10]. 

The underlying pathophysiology is related to reduced food intake and delayed gastric emptying induced by GLP-1 receptor agonists. Together with associated side effects such as nausea and vomiting, this can precipitate a state of starvation. Prolonged fasting and reduced carbohydrate intake subsequently promote lipolysis and ketone body production, ultimately resulting in starvation ketoacidosis [4]. In our case, the chronological sequence of events supports a possible causal relationship: initiation of semaglutide was followed by progressive gastrointestinal symptoms, ultimately leading to emergency presentation, with exclusion of alternative etiologies leading to the diagnosis of euglycemic starvation ketoacidosis. The underlying pathophysiology also explains the rapid resolution of symptoms and metabolic disturbance after receiving IV dextrose. All three previous cases describe young women (21-40 years old) with no significant medical history presenting to the emergency room with reduced oral intake and vomiting. In the case by Singh et al. describing tirzepatide use, the patient started with 5 mg once weekly (instead of the recommended 2.5 mg) and presented only three weeks after the first dose [6,9]. Our patient may also have initiated semaglutide at a higher-than-recommended starting dose (presumably 0.5 mg instead of 0.25 mg), with symptom onset occurring within approximately 10 days. Nevertheless, the precise dose remains uncertain, as the medication was obtained from an unregulated source and may have been counterfeit. GLP-1 receptor agonist side effects are known to be dose-dependent [1], which may have contributed to the development of euglycemic ketoacidosis in this setting. The case by Alghamdi et al., describing liraglutide use, states that their patient was taking the medication without a prescription [10]. This parallels our case, in which too, the patient obtained semaglutide without medical supervision. The use of GLP-1 receptor agonists in an unmonitored setting could be a significant contributing factor in the development of euglycemic ketoacidosis, particularly when early symptoms are not promptly identified. Alternatively, the case by Sood et al. described a patient using the medication under medical supervision for seven months, with a correct dose titration, but there was a superposition of a viral gastroenteritis [8]. 

Aside from the successful introduction among medical professionals, GLP-1 agonists have become a popular topic in media outlets and celebrity endorsements. This has no doubt influenced the illegal market of GLP-1 online sourcing. Regulatory agencies such as the European Medicines Agency (EMA) have issued warnings for falsely labeled pens being sold online, indicating they may not contain the claimed active substance and may contain harmful levels of other substances [11].

A recent study evaluating the safety of purchasing semaglutide online without a prescription found that approximately 42% of websites were associated with illegal pharmacy operations. Additionally, three semaglutide products purchased through these sources were tested, revealing that the actual semaglutide content significantly exceeded the labeled amount by up to 39% [12]. This indicates that patients using these products may unintentionally receive a substantially higher dose per injection than intended. In our patient’s case, no toxicological or compositional analysis was performed, so the administered dose cannot be accurately quantified, and the presence of potentially harmful contaminants cannot be excluded. Although speculative, an uncertainty in dosing and product quality may have contributed to the development of more severe adverse effects.

The adverse effects of these medications must always be weighed against the potential health risks associated with obesity. However, this risk-benefit balance was not applicable in our patient, whose baseline BMI was 26 kg/m². As highlighted in this report, euglycemic ketoacidosis represents one of these potential risks.

## Conclusions

This case highlights a clinically significant case of euglycemic starvation ketoacidosis occurring after off-label use of a GLP-1 receptor agonist for weight management. While GLP-1 agonists are generally well tolerated, this case highlights that their gastrointestinal adverse effects may, under certain conditions, precipitate starvation and subsequent ketoacidosis in susceptible individuals without diabetes. In addition, the growing availability of unregulated online pharmacies, often providing products of uncertain purity and dosing, may further increase the risk of such adverse events. However, causality regarding semaglutide itself, including the potential contribution of supratherapeutic dosing or product quality, cannot be established based on this single case study. Further systematic investigation is needed to better define the frequency, mechanisms, and predisposing factors for this rare but potentially serious complication. As the use of these agents continues to expand, it is essential for clinicians to remain vigilant and to ensure prompt and accurate diagnosis.
